# A commentary on “Effects of dyclonine mucilage and compound lidocaine cream as tracheal catheter lubricant on postoperative pharyngeal complications after general anesthesia”

**DOI:** 10.1097/JS9.0000000000003117

**Published:** 2025-07-23

**Authors:** Fu-Shan Xue, Dan-Feng Wang, Xiao-Chun Zheng

**Affiliations:** a Department of Anesthesiology, Shengli Clinical Medical College of Fujian Medical University, Fuzhou University Affiliated Provincial Hospital, Fujian Provincial Hospital, Fuzhou, China; b Department of Anesthesiology, Beijing Anzhen Hospital, Capital Medical University, Beijing, China

*Dear Editor*,

By conducting a single-center, randomized controlled trial with 90 patients who underwent laparoscopic cholecystectomy, Li *et al*^[1]^ demonstrated that compared with dyclonine mucilage, the use of compound lidocaine cream as the tracheal tube lubricant significantly reduced the incidence of postoperative sore throat (POST), and improved patient satisfaction. However, we noted several issues on the methodology and results of this study and would like to obtain the authors’ responses.

First, as the primary outcome of this study, Li *et al*^[1]^ did not specify the patient’ status when evaluating POST, though it is less severe at rest than during swallowing^[2]^. The participants underwent laparoscopic cholecystectomy and the occurrence of POST was assessed at 1, 6, and 24 h postoperatively, but the readers were not provide if a consistent postoperative multimodal analgesia scheme was given for all patients. Most important, the time relationship between assessment of POST and administration of postoperative analgesics was also unclear. In fact, the medications commonly recommended for postoperative multimodal opioid-sparing analgesia scheme in the current context of enhanced recovery after abdominal surgery, such as corticosteroids, paracetamol, non-steroidal anti-inflammatory drugs, N-methyl-D-aspartate receptor antagonists and α_2_-adrenergic receptor agonists^[3]^, are effective for symptom abatement and treatment of POST^[4]^. We are concerned that these unknown factors would have confused the assessment of the primary outcome.

Second, Li *et al*^[1]^ did not clearly state the occurrence of poor glottic visualization, difficult tube advancement and multiple attempts during the tracheal intubation, though they are the known risk factors of POST^[3]^. To determine effect of one intervention on primary outcome in a randomized controlled trial, all of the other factors that may influence the assessment of primary outcome must be standardized for avoidance of potential biases. Given that the study by Li *et al*^[1]^ is a randomized controlled trial with the small sample, we believe that the between-group balances of these potential confounders would be important to correctly interpret primary finding.

Third, in the results, Li *et al*^[1]^ reported that the incidence of POST at 1 and 6 h postoperatively significantly lower in the participants assigned into the compound lidocaine cream group than in those assigned into the dyclonine mucilage group. According to the results of table 2 of Li *et al*’ article^[1]^, we noted that the P values of total comparisons for the incidence of POST at 1 h (*P* = 0.097) and 6 h (*P* = 0.081) postoperatively between the compound lidocaine cream and dyclonine mucilage groups were larger than the designed level of statistically significant, that is, a *P* vale of less than 0.05. Li *et al*^[1]^ reported the incidences of POST with 0–3 scores at different time points, but not the total POST scores of all studied groups at each time point. Especially, this study did not compare the between-group differences in the incidences of POST with 0–3 scores at each time point. Because of lacking these data, an important question that this study cannot answer is whether the severity of POST at each time point is significantly different among three studied interventions.

Finally, in the methods, Li *et al*^[1]^ stated that postoperative patient satisfaction was evaluated based on the method described in a previous work of Bennett *et al*^[5]^. However, the article of Bennett *et al*^[5]^ does not include a detailed assessment of patient satisfaction. In the results, Li *et al*^[1]^ reported the proportions of patients with satisfaction scores of 0–4, but did not provide the patient response corresponding to each point score. For example, 0 means very satisfied and 4 indicates extremely dissatisfied, as described in other works assessing patient satisfaction by a 5-point Likert scale^[6,7]^. We argue that clarifying this issue will improve the transparency of this research design.

We ensure that our article is compliant with the TITAN Guidelines 2025, as shown in TITAN Guideline Checklist 2025^[8]^.

## Data Availability

All data is available.

## Research registration unique identifying number (UIN)

Not necessary.
